# Resolving the Taxonomy of Mountain Syrphidae (Diptera) in the Iberian Peninsula: The Species Group of *Cheilosia melanura* Becker, 1894

**DOI:** 10.3390/insects15090640

**Published:** 2024-08-26

**Authors:** Iván Ballester-Torres, Zorica Nedeljković, Pablo Aguado-Aranda, Ante Vujić, María Ángeles Marcos-García, Antonio Ricarte

**Affiliations:** 1 Research Institute CIBIO (Centro Iberoamericano de la Biodiversidad), Science Park, University of Alicante, Ctra. San Vicente del Raspeig s/n, San Vicente del Raspeig, 03690 Alicante, Spain; zoricaned14@gmail.com (Z.N.); pablo.aguado@ua.es (P.A.-A.); marcos@ua.es (M.Á.M.-G.); ricarte24@gmail.com (A.R.); 2 Department of Biology and Ecology, Faculty of Sciences, University of Novi Sad, Trg Dositeja Obradovića 2, 21000 Novi Sad, Serbia; ante.vujic@dbe.uns.ac.rs

**Keywords:** hoverfly, new species, COI, Sierra Nevada, integrative taxonomy

## Abstract

**Simple Summary:**

Hoverflies are a diverse group of dipterans (6800+ species worldwide) of high relevance to pollination and the regulation of insect pest populations. With nearly 500 described species worldwide, *Cheilosia* represents the largest hoverfly genus in the Palaearctic, with larvae of most species feeding on flowering plants or fungal tissues. The complexity of this genus is eased by the definition of various species groups, including the group of *Cheilosia melanura*, which is assessed in this paper in the context of the Iberian Peninsula. The aim of the present work is to improve the knowledge of this species group by following an integrative approach, i.e., a combined use of different data sources (e.g., morphology and genetics) to solve a taxonomic problem or evaluate a taxonomic scenario. With this approach, we found and described a species new to science from Spain and provided the first identification key for all Iberian species of the group, including most of the European species.

**Abstract:**

The largest genus of Palaearctic Syrphidae, *Cheilosia* Meigen, 1822 (Syrphidae: Rhingiini), is currently under revision in the Ibero-Balearic region (Iberian Peninsula + Balearic Islands). Prior to this study, various species groups with putative phylogenetic support were defined for this genus of complex taxonomy. The group of *Cheilosia melanura* Becker, 1894 includes species distributed all over Europe, with some of these species being similar each other in both morphology and genetics. After recent fieldwork in different Iberian localities and consultation of entomological collections, a new species from Sierra Nevada (Granada, Spain) was uncovered, described, and illustrated. Data on diagnostic characters, intraspecific variability, and adult biology were also provided. Maximum likelihood analyses of the fragment “C” of the 5′ end of the cytochrome *c* oxydase subunit I (COI-5′) and complete COI-5′ were performed to explore and support morphological species concepts within the group. The Spanish-endemic *Cheilosia andalusiaca* Torp Pedersen, 1971 is recognised here to be part of the *C. melanura* group based both on morphological and molecular evidence. *Cheilosia carbonaria* Egger, 1860 and *Cheilosia cynocephala* Loew, 1840 from the Iberian Peninsula are reported for the first time based on specimens originating in the Spanish Pyrenees. An identification key for the Iberian species of the *C. melanura* group is provided.

## 1. Introduction

With nearly 500 species, *Cheilosia* Meigen, 1822 (Syrphidae: Eristalinae) is the largest hoverfly genus in the Holarctic Region [1,2]. Adults are usually dark flies, with a shiny thorax and abdomen and body hairs ranging from black to yellow, sometimes reddish [3]. Larvae are ‘short-tailed’; those of most species are phytophagous, and just a few are saprophagous in association with trees [4,5]. Regarding adult classification, there have been various attempts to split *Cheilosia* into different morphological species groups in order to ease the understanding of such a complex genus comprising high levels of species richness [6], cryptic speciation [3], and intraspecific variability [7]. The group of *Cheilosia melanura* Becker, 1894 is one of these morphological groups that integrates species with antennae separated by the lunule, hairy eyes (at least on the upper half), a well-expressed mouth edge, a scutellar margin with or without black bristles, legs with tibiae that are entirely pale, pale at both ends, or completely dark, sternites that are usually shining; male genitalia that are similar in shape among the species of the group [8], with a short and rounded surstylus, an elongated ventral lobe of the gonostylus that widens towards the apex, and a short dorsal lobe of the gonostylus, where both lobes are of approximately the same width.

In their assessment of the complex of *Cheilosia vernalis* (Fallén, 1817), Ståhls et al. [3] included the following species in the *C. melanura* group: *Cheilosia bergenstammi* Becker, 1894, *Cheilosia brachysoma* Egger, 1860, *Cheilosia bracusi* Vujić and Claussen, 1994, *Cheilosia carbonaria* Egger, 1860, *Cheilosia chloris* (Meigen, 1822), *Cheilosia cynocephala* Loew, 1840, *Cheilosia fraterna* (Meigen, 1830), *Cheilosia lenis* Becker, 1894, *Cheilosia lenta* Becker, 1894, *C. melanura*, *Cheilosia rhynchops* Egger, 1860*, Cheilosia sootryeni* Nielsen, 1970, and *C. vernalis*. Although Ståhls et al. [3] recognised the similarity between *C. vernalis* and *C. reniformis* Hellen, 1930, they did not explicitly regard *C. reniformis* as a part of the *C. melanura* group. The same authors included *Cheilosia rufimana* Becker, 1894 as part of the *C. melanura* group, but later on, Vujić et al. [9] placed this species in the group of *Cheilosia proxima* (Zetterstedt, 1843). None of these species are currently present in North Africa [6].

Although some works try to shed light on the taxonomy of the *C. melanura* group [10,11], some species are still known to have a difficult delimitation. For example, species of the *C. melanura* group are considered to have the least differentiated male genitalia within the genus [12], while certain species, such as *C. vernalis,* appear to form a complex of morphologically similar species that require further molecular data to resolve [3]. Moreover, intermediate forms are known to occur between some species [13], such as for the pair *C. vernalis*/*C. reniformis,* which sometimes display overlapping morphological features even though genetics support a separation between them [3].

Nowadays, only five species of the *C. melanura* group (*C. bergenstammi*, *C. bracusi*, *C. chloris*, *C. fraterna*, and *C. vernalis*) have been recorded in Spain [14], all of them from the northern half of the mainland territory. Three species, *C.* aff. *fraterna*, *C. fraterna,* and *C. vernalis*, were reported in mainland Portugal [15,16]. In the frame of this work, some individuals supposed to belong to the *C. melanura* group were found in Sierra Nevada (southern Spain). After a preliminary examination, these specimens displayed a unique combination of morphological traits differing from the rest of the known species in the group. Further specimens of the *C. melanura* group were added to the results after fieldwork in other Iberian localities. Thus, the objectives of the present work are (a) to assess the diversity of the *C. melanura* group in the Iberian Peninsula and (b) to explore the systematic position of the studied species based on molecular evidence.

## 2. Materials and Methods

### 2.1. Study Area and Examined Material

**Figure 1:**
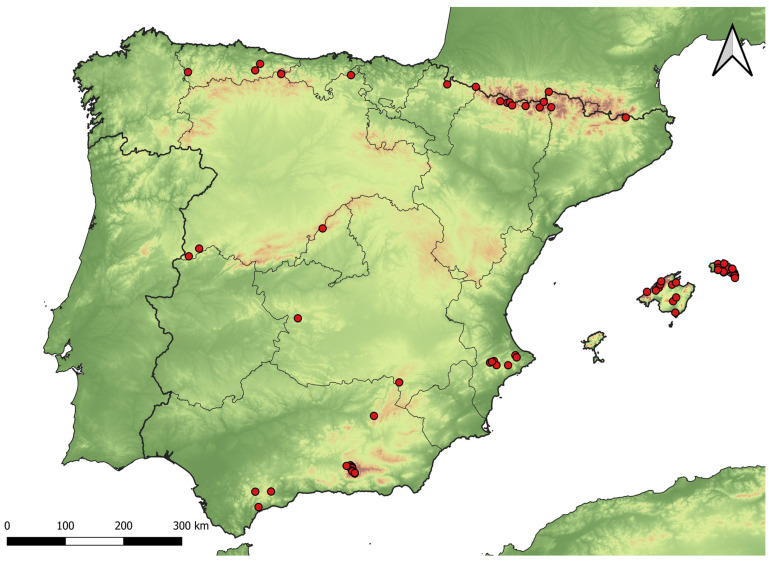
Map of the localities (red circles) sampled between 2021 and 2024 in the Iberian Peninsula and the Balearic Islands.

Located in southwestern Europe, the Iberian Peninsula has an extension of 583,254 km^2^ and a very contrasting and uneven topography, with mountain ranges that are mainly distributed from west to east [17]. Various areas in these mountain ranges all over the Iberian Peninsula were sampled between 1980 and 2024, including the western ‘Sistema Central’ (province of Salamanca), ‘Cordillera Cantábrica’ (province of León, regions of Asturias and Cantabria), and the western (province of Navarra), central (province of Huesca), and eastern Pyrenees (province of Girona). Major sampling efforts were carried out between 2021 and 2024 in four National Parks: ‘Parque Nacional de Ordesa y Monte Perdido’ (central Pyrenees, province of Huesca), ‘Parque Nacional de Picos de Europa’ (northern Spain, province of León), ‘Parque Nacional de la Sierra de Guadarrama’ (central Spain, province of Madrid), and ‘Parque Nacional (also Natural Park) de Sierra Nevada’ (southern Spain, province of Granada) (Figure 1). The corresponding permits were obtained for sampling in all mentioned protected areas.

**Figure 2:**
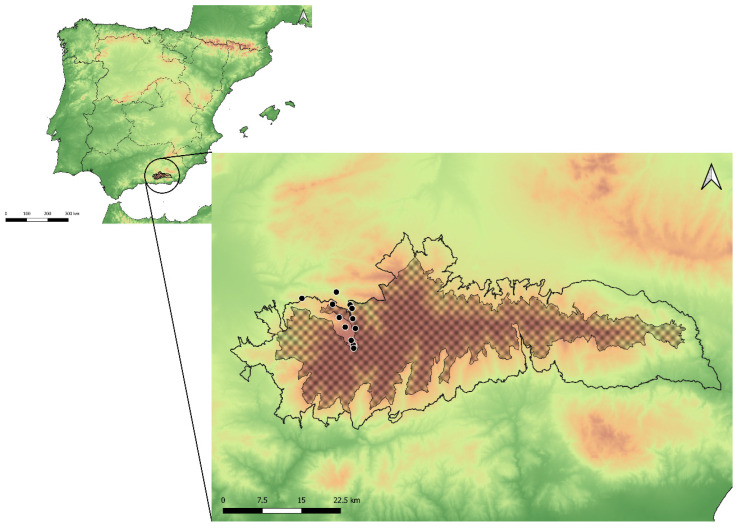
Location of the Sierra Nevada and distribution of the sampled localities (black circles) in the present *Cheilosia* study. The outer black line represents the border of the Sierra Nevada Natural Park, whilst the darkened area corresponds with the National Park area.

The Sierra Nevada belongs to the Baetic System, a mountain range extending along most of southwestern Spain. With over 850 km^2^ of protected area, Sierra Nevada National Park has a wide altitudinal range, from 300 m at the east end of the park to the Mulhacén, the highest peak of the Iberian Peninsula (3478 m asl) [18]. Sierra Nevada is an exceptional refuge of flora and fauna on the European continent due to its historical conditions (its strategic biogeographical location in the west of the Mediterranean region), its geographical isolation, the abruptness of ecological gradients (with its wide altitudinal range), and the diversity of ecological niches. It has more than 2100 catalogued plant species, 116 of which are threatened [19]. In the frame of the present study, 12 northwestern localities of the Sierra Nevada ranging from 1400 to 3000 m asl were surveyed in 2021 and 2022 (Figure 2).

A total of 20 specimens belonging to a supposedly new species were collected inside the protected area of the Sierra Nevada mountain range. This putative new species was compared with individuals of other species of the *C. melanura* group (indicated in the ‘Other species of the *Cheilosia melanura* species group’ section). All examined material, whether or not it is Iberian, is deposited in the following collections:

- AUC = Alberto Uría Personal Collection
- AVPC = André Van Eck Personal Collection
- CEUA-CIBIO = ‘Colección Entomológica de la Universidad de Alicante’, Spain
- FSUNS = Department of Biology and Ecology, Faculty of Sciences, University of Novi Sad, Serbia
- FVMC = Frank Van de Meutter Personal Collection
- MCNB = Museu de Ciències Naturals de Barcelona
- MNCN = Museo Nacional de Ciencias Naturales, Madrid
- UNAV = Colección de la Universidad de Navarra
- XLPC = Xavier Lair Personal Collection

To avoid unnecessary repetition, the collection acronym is mentioned only when the material is found in a collection other than the CEUA-CIBIO (except for holotypes and paratypes of the new species). All specimens from the CEUA-CIBIO bear a barcode label with a unique identifier, which is also indicated in the list of examined materials. Most specimens were collected with a hand net, and the remainder were collected with Malaise traps (‘T. Ma.’ in the text).

When presenting the new species’ information, the following protocol is used: diagnosis, etymology, examined material, description (based on the male holotype), female description (based on a paratype), intraspecific variation, distribution, and biology. The morphological terminology follows that of Thompson [20], except for the use of “hairs” instead of “pile”. For the hind tibia colour, the term ‘incomplete’ is used to indicate that a certain section of an area becomes lighter but does not totally change its colour; for example, it applies to a ring-shaped area that is not totally homogeneous in colour. The terminology referring to the male genitalia follows that of Clauβen [21]. Specimens were studied under a Leica© M80 binocular stereomicroscope (Leica Camera AG, Wetzlar, Germany). Photographs and specimen measurements were taken with a Leica© DFC 450 camera attached to a Leica© M205 C binocular microscope (Leica Camera AG, Wetzlar, Germany) using the Leica© Application Suite X v.5.1.0.25593 (LAS X) software (Leica Microsystems, Wetzlar, Germany). Measurements are indicated in Figure 7, with the following abbreviations: bl = body length; bw = body width; ec = eye contiguity length; ft = frontal triangle length; wl = wing length. Male genitalia were cleared and prepared following Ricarte et al. [22] and stored in polyethylene microvials with glycerol. Male genitalia were drawn in the Photoshop© 2021 software (ADOBE, San Francisco, CA, USA) using an XP-PEN© Artist 13.3 Pro digital tablet (XPPEN TECHNOLOGY Co., Shenzhen, China) from stacks of photos produced with the above-mentioned microscope equipment and software. Maps were produced with the QGIS 3.28.10 software [23].

### 2.2. Molecular Analyses

A total of 14 specimens were used to sequence the 5′ end region cytochrome c oxydase subunit I (COI-5′ hereafter). DNA was extracted from both the right mid- and hind leg of single pinned individuals using the NZY gDNA Isolation Kit while following the manufacturer’s protocol for animal tissues. PCR amplification of the COI-5′ gene was performed with the universal and custom primers used by Aguado-Aranda et al. [24]. The COI-5′ fragments “A”, “B”, and “C” were amplified for specimens where the entire COI-5′ was not successfully amplified. PCR reactions were performed in 25 µL reactions containing 1 × of Buffer reaction, 0.4 mM of dNTPs, 0.2 µM of each primer, 0.65–2 mM of MgCl_2_, and 1–2 units of DNA polymerase. The thermocycler conditions followed those used by Ståhls and Barkalov [25] but with annealing between 49 and 50 °C and with 29–40 cycles, except for CEUA_S402, S403, and S408, for which PCR consisted of an initial denaturalisation for 3 min at 94 °C, 45 cycles of 45 s at 94 °C, and annealing for 45 s at 45 °C and 1 min at 72 °C, with a final elongation step of 5 min at 72 °C. All PCR products were visualised with an electrophoresis process in a 2% agarose gel and sequenced at Macrogen Inc. (Macrogen, Spain), except for CEUA_S408, which was sequenced in ‘Servicios Técnicos’, University of Alicante, Spain.

The COI-5′ barcode sequences obtained were cleaned and edited by eye with the Sequencher v5.4.6 program (Gene Codes Corporation 2017, Ann Arbor, MI, USA). COI-5′ sequences for selected species of *Cheilosia* available at the public GenBank and BOLD repositories were downloaded (Table 1). Sequence alignment was automatically performed and checked with AliView v1.27 [26]. Phylogenetic analyses were carried out under Maximum Likelihood (ML hereafter) frameworks in MEGA7 [27] with 1000 bootstrap replications using the General Time-Reversible (GTR) model with the gamma distribution for the full COI-5′ matrix and for fragment “C” of the COI-5′ matrix. The resulting trees were rooted based on a *Ferdinandea cuprea* (Scopoli, 1763) sequence. All species of the group that are present on the Iberian Peninsula were included, except for *C. chloris*, as no fresh material was available for molecular analyses, and *C. vernalis*, as no DNA from fresh specimens was sequenced due to the molecular complexity of the species [3] and the availability of COI-5′ sequences in the genetic databanks. To complete the phylogenetic trees, we used sequences available in GenBank and BOLD from other European countries. COI-5′ sequences from part of the *C. melanura* species group were also obtained from the above-mentioned databases. Species of the group that were not represented in the phylogenetic trees had no available COI-5′ sequences online. For some of the studied specimens (*C. andalusiaca*), we were only able to correctly amplify fragment “C”. Therefore, a Maximum Likelihood tree including only this fragment was also created.

## 3. Results

### 3.1. Integrative Approach

**Figure 3:**
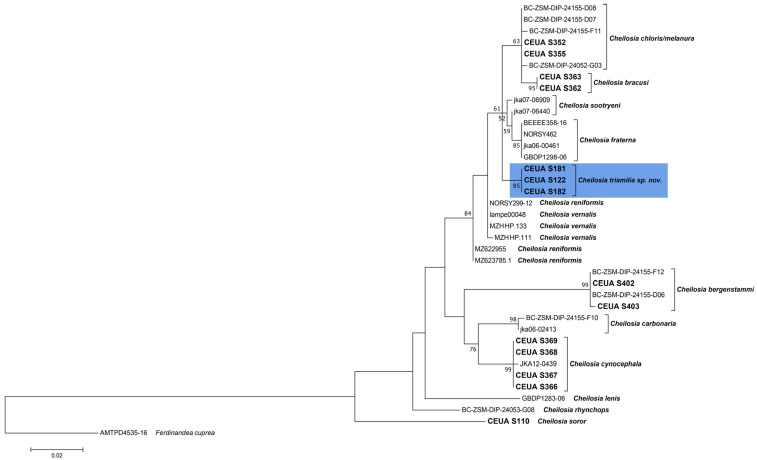
Maximum Likelihood tree based on COI-5′ of Iberian and European specimens of the *Cheilosia melanura* group. DNA vouchers for own sequences are highlighted in bold. DNA vouchers for the new species are grouped in a blue rectangle. Bootstrap values of >50 are shown near nodes. Branch lengths are measured in numbers of substitutions per site.

**Figure 4:**
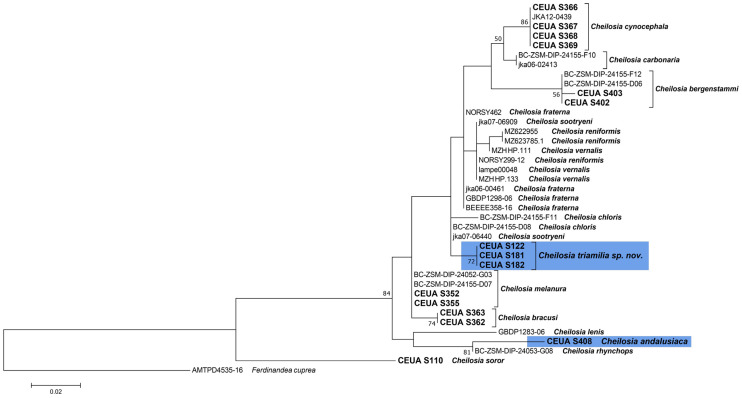
Maximum Likelihood tree based on fragment “C” of COI-5′ of Iberian and European specimens of the *Cheilosia melanura* group. DNA vouchers for own sequences are highlighted in bold. DNA vouchers for the new species and *Cheilosia andalusiaca* are each grouped in a blue rectangle. Bootstrap values of >50 are shown near nodes. Branch lengths are measured in numbers of substitutions per site.

As a result of the revision of the specimens in the above-mentioned collections and recent field surveys, 235 specimens of the following species were studied: 7 specimens of *C. andalusiaca* (3 males and 4 females), 33 specimens of *C. bergenstammi* (19 males and 14 females), 1 female of *C. brachysoma*, 39 specimens of *C. bracusi* (33 males and 6 females), 6 specimens of *C. carbonaria* (2 males and 4 females), 3 specimens of *C. chloris* (2 males and 1 female), 9 specimens of *C. cynocephala* (4 males and 5 females), 38 specimens of *C. fraterna* (19 males and 19 females), 22 individuals of *C. aff. vernalis* (13 males and 9 females), 7 specimens of *C. lenis* (3 males and 4 females), 1 male and 1 female of *C. lenta*, 12 specimens of *C. melanura* (6 males and 6 females), 1 male and 1 female of *C. rhynchops*, 52 specimens of *C. vernalis* (32 males and 20 females), and 1 male and 1 female of *Cheilosia “aff. fraterna”* sensu [16]. In the molecular analyses, 39 COI-5′ sequences were used to develop the phylogenetic trees (Figure 3 and Figure 4). As we were only able to obtain the “C” fragment of COI-5′ for *C. andalusiaca*, we built a tree with only the “C” fragments of all the sequences indicated in Table 1 (Figure 4). Both COI-based trees showed high bootstrap values (>70) for the new species that clustered in a single clade (Figure 3 and Figure 4), supporting our morphological hypotheses. Therefore, based on both morphological and molecular evidence, the new species was characterised.

### 3.2. New Species Description

**Figure 5:**
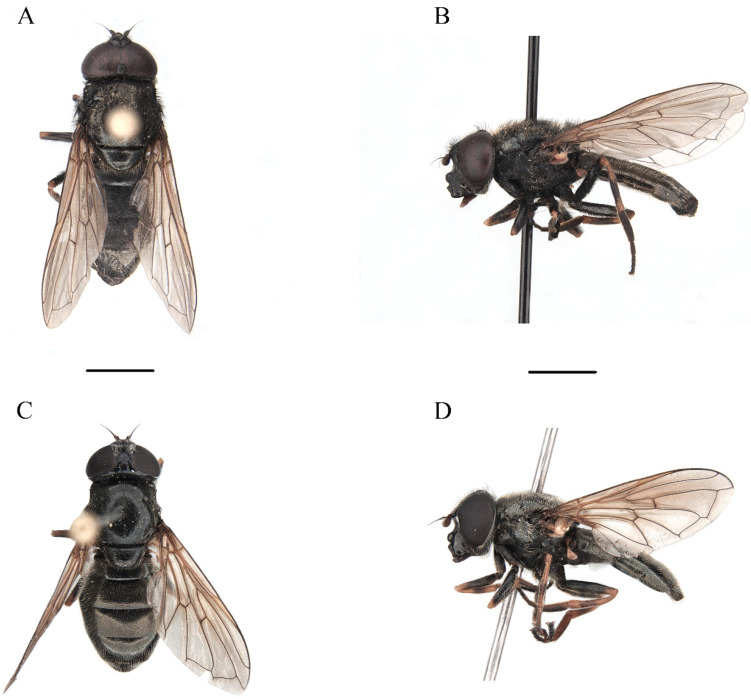
*Cheilosia triamilia* Ballester-Torres, Ricarte, and Nedeljković sp. nov. (**A**) Holotype, male, dorsal view. (**B**) Holotype, male, lateral view. (**C**) Paratype (CEUA00114370), female, dorsal view. (**D**) Paratype, female, lateral view. Scale bars: (**A**,**C**) 2.5 mm; (**B**,**D**) 2.5 mm.

**Figure 6:**
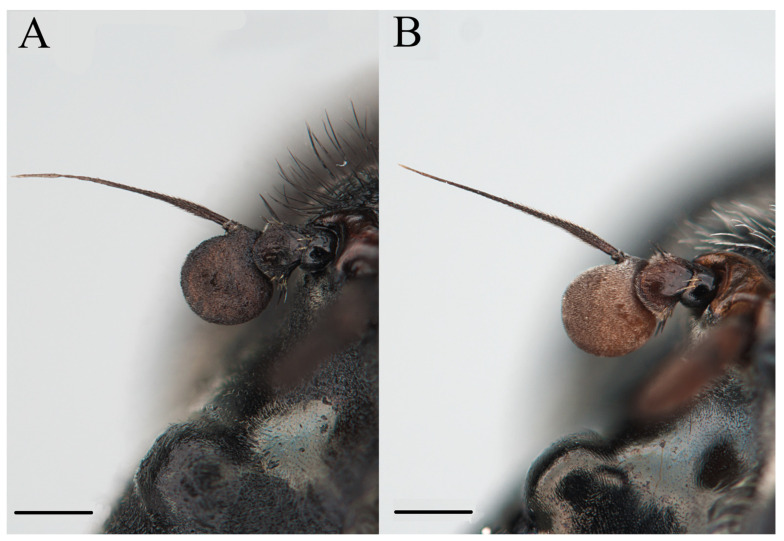
*Cheilosia triamilia* Ballester-Torres, Ricarte, and Nedeljković sp. nov. (**A**) Holotype, male, antenna, lateral view. (**B**) Paratype, female, antenna, lateral view. Scale bars: (**A**,**B**) 0.25 mm.

**Figure 7:**
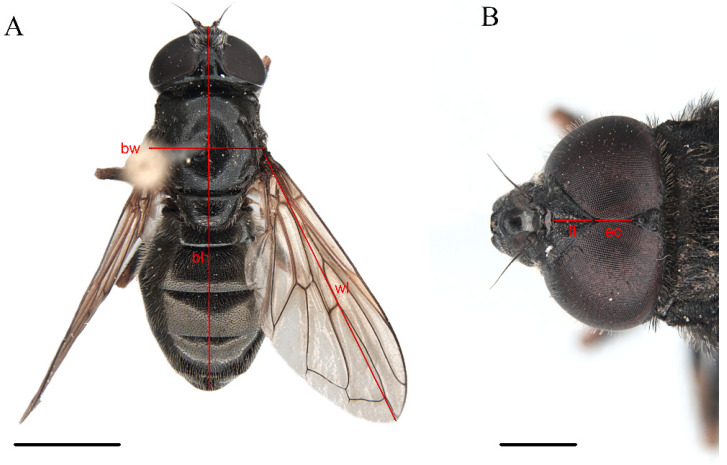
*Cheilosia triamilia* Ballester-Torres, Ricarte, and Nedeljković sp. nov.; measurements of the described characters (**A**) Paratype, female. (**B**) Holotype, male. Abbreviations: bl = body length; bw = body width; ec = eye contiguity length; ft = frontal triangle length; wl = wing length. Scale bars = (**A**) 2.5 mm; (**B**) 1 mm.

**Figure 8:**
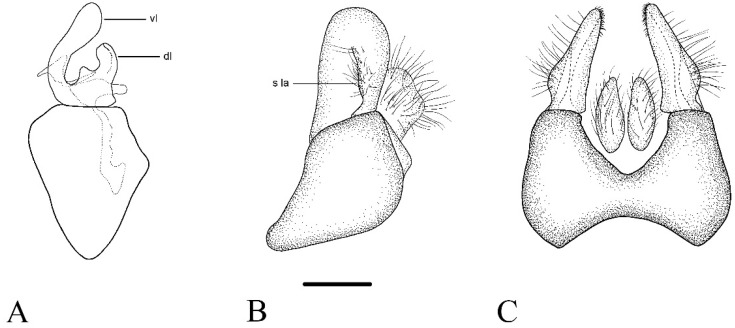
*Cheilosia triamilia* sp. nov., male genitalia, holotype. (**A**) Hypandrium with right gonostylus, lateral view. (**B**) Epandrium with right surstylus, lateral view. (**C**) Epandrium, dorsal view. Abbreviations: dl: dorsal lobe of gonostylus; s la: surstylus lamella; vl: ventral lobe of gonostylus. Scale bar (**A**–**C**): 250 µm.

After morphological and molecular analyses of fresh specimens collected in the Sierra Nevada (Granada), a new species was uncovered and described. This species belongs to the subgenus *Cheilosia* s. str. Barkalov, 2002 and the *C. melanura* species group (Milankov et al., 2002 [8]).
Class Insecta Linnaeus, 1758Order Diptera Linnaeus, 1758Family Syrphidae Latreille, 1802Subfamily Eristalinae Newman, 1834Tribe Rhingiini Newman, 1834Subtribe Cheilosiina Williston, 1885Genus ***Cheilosia*** Meigen, 1822Subgenus *Cheilosia* s. str Barkalov, 2002***Cheilosia triamilia*** Ballester-Torres, Ricarte, and Nedeljković sp. nov. (Figure 5, Figure 6, Figure 7 and Figure 8) urn:lsid:zoobank.org:act:80468FDD-8508-48F0-9EE6-390596590A94.

#### 3.2.1. Diagnosis

Medium-sized black species with ‘stocky’ abdomen, sparsely-haired lower fourth of eye, entirely black or basally red, round basoflagellomere, slightly thickened basal part of arista, yellow hind tibia with a faint or incomplete black ring, and tarsi of all legs dorsally black.

#### 3.2.2. Etymology

The specific epithet ‘triamilia’ is an invariable word meaning ‘three thousand’ in Latin. It refers to the approximate altitude of the locality where this species was first collected.

#### 3.2.3. Examined Material

##### Holotype

SPAIN · ♂; Granada, Sierra Nevada, Monachil, Pradollano, Estación de esquí, 2180 m asl; 29/V/2022; Z. Nedeljković leg.; CEUA00114353; CEUA-CIBIO.

##### Paratypes

SPAIN · 1♂; same locality and altitude as for holotype; 26/V/2022; I. Ballester-Torres leg.; CEUA00114354; CEUA-CIBIO · 3♂♂; Granada, Sierra Nevada, Güéjar Sierra, El Dornajo, Parking camino a Peña del Perro, 1895 m asl; 27/V/2022; I. Ballester-Torres and P. Aguado leg.; CEUA00114355 to 00114357; CEUA-CIBIO · 1♂ and 1♀; same locality and altitude as for the preceding; 29/V/2022; P. Aguado leg.; CEUA00114358 and 00114359; CEUA-CIBIO · 5♂♂ and 1♀; same locality, altitude, and date as for the holotype; Z. Nedeljković, I. Ballester-Torres, and P. Aguado leg.; CEUA00114360 to 00114365; CEUA-CIBIO · 1♂ and 4♀♀; Granada, Sierra Nevada, Monachil, Museo Fuente Alta, 2025 m asl; 30/V/2022; I. Ballester-Torres and P. Aguado leg.; CEUA00114366 to 00114369, 00114480; CEUA-CIBIO · 2♀♀; Granada, Sierra Nevada, Monachil, camino a Laguna de Las Yeguas, 2890 m asl; 25/VI/2021; A. Ricarte and I. Ballester-Torres leg.; CEUA00111316 and 00114370; CEUA-CIBIO · 1♂ and 1♀; Capileira, Rio Mulhacén, 2450 m asl, 37.030N, −3.327E; 18/IV/2023; F. Van de Meutter Leg.; FVMC.

#### 3.2.4. Description

**Male**. MEASUREMENTS (Figure 7A,B) (mm). Holotype: bl = 9.29; bw = 3.08; wl = 7.55.

HEAD. Eye with dark brown hairs dorsally, the reminder of eye pilosity lighter. Eye pilosity dense, except for the sparsely-pilose lower fourth of eye. Eye hairs longer than the anterior ocellus’ maximum diameter, but shorter than the vertical triangle hairs. Occiput narrow on its dorsal half, wider ventrally. Occiput pollinosity greyish white dorsally, whiter towards the lower part of the occiput. Occiput with yellowish brown hairs and a few long black hairs intermixed on its dorsal third. Vertical triangle black, faintly grey-pollinose posteriorly. Ocellar triangle slightly elevated in lateral view, with punctured surface, ocelli very close to eye margins (distance shorter than an ommatidium diameter). Vertical triangle with long, erect, black hairs, with their tips forwardly inclined, and some shorter, yellow hairs intermixed. Frontal triangle black, punctured, faintly pollinose on eye margins, with a conspicuous medial sulcus. Frontal triangle slightly convex in lateral view, with long, erect, black hairs, longer than those on eye. Frontal triangle approximately as long as the eye contiguity. Lunule shiny reddish brown, with a forward extension separating the antennal sockets. Scape and pedicel with black hairs. Scape black. Pedicel black, brown on the apical part of its outer side. Basoflagellomere round, black and faintly white pollinose. Arista 2.5 × longer than the basoflagellomere height, black, slightly thickened basally, with short (less than half the maximum width of arista) hairs all over its length. Face black, with greyish-white microtrichia, especially dense below the antennal insertions and sparsely microtrichose near the mouth. Face with a well-developed facial tubercle and a mouth edge protruding beyond the level of the facial tubercle. Parafacia wide, at its maximum width 0.8 × longer than the basoflagellomere width. Parafacia faintly grey pollinose, with yellowish hairs, nearly as long as the eye hairs. Gena shiny, without microtrichia.

THORAX. Scutum and scutellum shiny black, with bronze reflections. Scutum grey pollinose on the anterior margin. Anterior half of scutum with yellow and black hairs intermixed, posterior part only with black hairs medially. Scutellum with long (approximately 0.5 × the scutellum length), erect, black hairs, intermixed with some long yellow hairs mainly on the lateral part, without setae. Scutellum with a conspicuous subscutellar fringe of yellow hairs, shorter than the longest hairs on the central plate of scutellum. Inner part of postpronotum pollinose. Anterior anepisternum bare. Posterior anepisternum, anterior anepimeron and katepisternum both with black and yellow hairs. Katepisternum with two groups of hairs widely separated by a bare gap. Katatergum with dense yellow fine pilosity. Katepimeron and meron bare. All coxae black and covered both in black and whitish hairs. All femora black except for their pale-reddish apices, with black hairs all over (shorter towards the femur apex) except on the basal part, where pale hairs intermix. Pro- and mesofemur with long black hairs postero-ventrally. Metafemur with conspicuous black spines ventrally. All tibiae pale with a conspicuous black ring centrally, except the hind tibiae (the black ring is incomplete on the ventral side). Protibia with pale hairs anteriorly and black hairs posteriorly. Mesotibia only with black hairs. Metatibia with black hairs anteriorly. Posterior side of the metatibia with yellowish white hairs except for the basal half where black hairs intermix. All tarsi black, except for the pale ventral side of the metatarsomeres 1–3. Claws pale on the basal half. Wing wholly microtrichose. Pterostigma brown pigmented. Base of wing plus cells C and SC faintly brown pigmented. Wing membrane just along veins CuA, basal half of R4+5 and r-m narrowly brown pigmented. Calypter white, yellowish marginally. Halter dark basally, with orange-brownish capitulum.

ABDOMEN. Oval, stocky (1.5 × longer than wide at its maxima), slightly wider than the scutum at the level of wing basis. Shiny black with bronze reflections. All terga pale haired. Terga II and III with a central patch of adpressed hairs, the patch on the tergum II covering a larger extension than that on the tergum III (the longest hairs of the lateral margins of abdomen found at the anterior corner of tergum II). All sterna shiny. Sterna I and II with erect pale hairs (hairs of sternum I inclined apically). Sterna III and IV with adpressed pale hairs. Pre-genital segments shiny, with faint greyish pollinosity and erect pale hairs.

GENITALIA (Figure 8A–C). Surstylus short and rounded (1.6 × longer than wide in lateral view). Lateral expansion of surstylus short, not surpassing half of the surstylus size. Ventral lobe of the gonostylus elongate, clearly widening towards the apex. Dorsal lobe of the gonostylus shorter, as wide as ventral lobe. Gonostylus with a protrusion between both lobes (1/3 of the dorsal lobe length).

**Female** (based on the paratype CEUA00114370). MEASUREMENTS (mm) (Figure 7A). Paratype (♀): bl = 8.59; bw = 2.90; wl = 6.79.

Same as the male except for the following characters: eye with more sparse hairs; frons shiny, without pollinosity, with semi-erect yellow hairs; facial microtrichia only present as a stripe below the antennal insertions, extending from eye to eye, including the dorsal part of parafacia; parafacia with an orange spot on ventral part, on the eye margin; basoflagellomere slightly larger; scutum and scutellum with short, semi-erect hairs; subscutellar fringe hairs longer than the longest hairs on the scutellum; posterior anepistermun, anterior anepimeron and katepisternum with only yellow hairs; coxae only with yellowish white hairs; femora with yellowish white hairs all over, except on the apical part of mesofemur (postero-ventrally); ventral black spines of metafemur shorter or almost absent; black ring of the hind femora almost indiscernible; hairs of all tibiae mainly pale, except for a line of black hairs posteriorly; all tarsi black, except for the pro- and mesotarsomeres 1–3, paler; metatarsomeres 1–2 black with both basal and apical parts paler; abdomen roundish (1.2 × longer than wide at its maxima), clearly wider than the scutum at the level of wing basis; central part of tergum IV with adpressed hairs; adpressed hair patch of terga II and III each of equal size; sterna II with erect adpressed hairs.

#### 3.2.5. Taxonomic Notes

*Cheilosia triamilia* sp. nov. has a distinct shape of both the ventral and dorsal lobes of the gonostylus, which are similar to those in *C. bergenstammi* and *C. fraterna* but differ in the shape and size of the bulge located between both gonostylus lobes. *Cheilosia triamilia* sp. nov. and *C. fraterna* have a pale hind tibia or an incomplete black ring (ventrally), which is sometimes very inconspicuous (rarely complete in *C. triamilia* sp. nov. but faint black). Both species can be separated by the eye hairs and the basoflagellomere colour. In *C. fraterna,* the lower half of the eye is bare and has an orange basoflagellomere, instead of the dark basoflagellomere and hairy eyes on the lower half in *C. triamilia* sp. nov. Females of *C. triamilia* sp. nov. are morphologically similar to those of *C. vernalis*, however, the eyes of *C. triamilia* sp. nov. have sparse hairs on the lower fourth, whilst females of *C. vernalis* have hairy eyes all over their surface. Nonetheless, this last character has proven somewhat variable because many specimens of *C. vernalis* bear sparsely hairy eyes or even virtually bare eyes ([3]). Black scutellar bristles are usually present in *C. vernalis* (some specimens may lack these bristles ([3])), while *C. triamilia* sp. nov. has no thick dark bristles on the posterior part of the scutellum. The black ring on the hind tibia is always present in *C. vernalis*, whilst in *C. triamilia* sp. nov., there is an incomplete or faint black ring.

#### 3.2.6. Variation

*Cheilosia triamilia* sp. nov. shows intraspecific variation in the basoflagellomere coloration (from brown with a reddish orange basal part to entirely black), size of the patch of black hairs on the posterior part of scutum, colour of katepisternum hairs (yellow with a few black to only yellow), intensity of the wing pigmentation and size and intensity of the hind tibia black markings (from hind tibia mainly pale to an incomplete dark ring, rarely complete).

#### 3.2.7. Distribution

**Figure 9:**
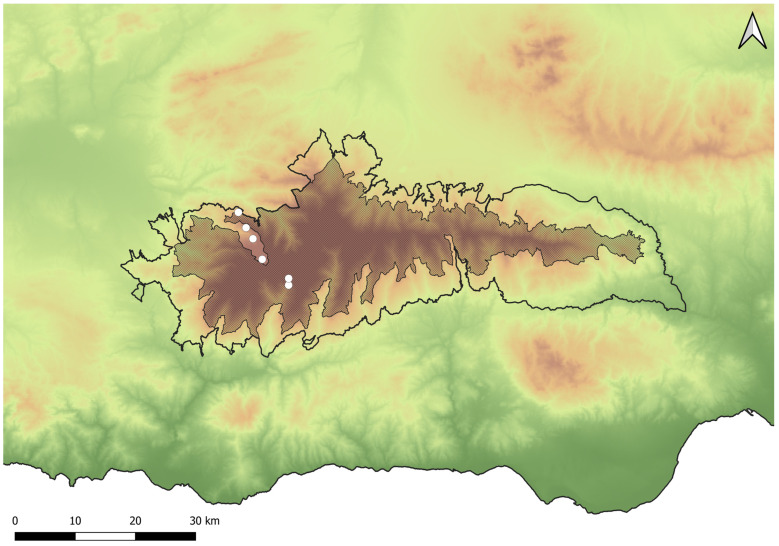
Distribution of *Cheilosia triamilia* sp. nov. in the Sierra Nevada mountain range (white dots). The outer black line represents the limits of the Natural Park of Sierra Nevada, whilst the darkened area corresponds with the National Park area.

This species was only recorded in the western part of the Sierra Nevada (Granada) (Figure 9) and is endemic to Spain.

#### 3.2.8. Biology

All individuals of *C. triamilia* sp. nov. were collected flying from late May to late June. Adults were found at a high altitudinal range (1885–2890 m) visiting flowers of *Ranunculus demissus* DC. (Ranunculaceae) and *Salix atrocinerea* Brot. (Salicaceae).

### 3.3. Examined Material of Other Species of the Cheilosia melanura Group

The materials of all other studied species examined with the purpose of the present taxonomic revision are listed below, including Iberian (indicated with an asterisk, *) and non-Iberian species of the *C. melanura* group. Examined material is also available as CSV format in Table S1.

*Cheilosia andalusiaca* Torp Pedersen, 1971*

**Figure 10:**
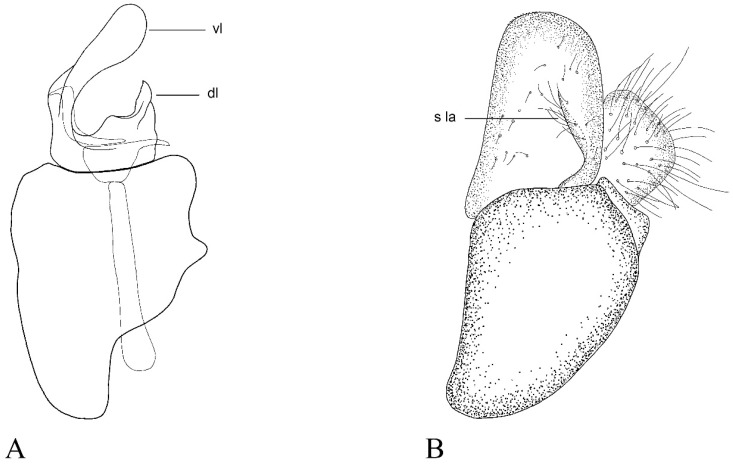
*Cheilosia andalusiaca* Torp Pedersen, 1971, male genitalia (based on CEUA00016731). (**A**) Hypandrium with the right gonostylus, lateral view. (**B**) Epandrium with the right surstylus, lateral view. Abbreviations: dl: dorsal lobe of the gonostylus; s la: surstylus lamella; vl: ventral lobe of the gonostylus. Scale bar: (**A**,**B**) 250 µm.

This is the first time that *C. andalusiaca* has been recognised as a member of the *C. melanura* group, and its male genitalia are illustrated here for the first time (Figure 10). The dorsal lobe of the gonostylus has a pointed tip, whilst most of the species of the *C. melanura* group have a rounded dorsal lobe of the gonostylus.

**Examined material. New. SPAIN ·** 1♀; Granada, Capileira, 1720 m asl, 37.004N −3.343E; 17/IV/2023; F. Van de Meutter Leg.; FVMC.

**Published. SPAIN ·** 1♂; Salamanca, Candelario; 09/VI/1982; C. Urones Leg.; CEUA00016729 · 1♂; Salamanca, La Honfría, Linares de Riofrío; 21/IV/1977; M.A. Marcos-García Leg.; CEUA00016731 · 2♀♀; Cáceres, Puerto del Torno, 950 m asl; 04/IV/1981; M.A. Marcos-García Leg.; CEUA00016732 and 00016734 · 1♀; Cáceres, San Martín de Trevejo, 900 m asl; 19/IV/1981; M.A. Marcos-García Leg.; CEUA00016733 · 1♂; León, Laguna de Arbás; 13/VI/1986; M.A. Marcos-García Leg.; CEUA00016730 (all specimens published in [28]).

*Cheilosia bergenstammi* Becker, 1894*

**Examined material. New. SPAIN ·** 1♀; Asturias, Espinaredo, Piloña; 28/VIII/2019; M.A. Marcos-García Leg.; CEUA00109094 · 1♀; León, Posada de Valdeón, Los Llanos, 43°9′23.6″ N 4°54′59″ O, 902 m asl; 27/VIII/2021; A. Ricarte and Z. Nedeljković Leg.; CEUA00111347 · 4♂♂ and 2♀♀; León, Posada de Valdeón, Pto. de Panderrueda, 43°7′31″ N 4°58′53.9’’ O, 1463 m asl; 27/VIII/2021; A. Ricarte and Z. Nedeljković Leg.; CEUA00111340, 00111344, 00111346, 00111348, 00111349, and 00111359 · 1♀; Cantabria, Puerto de Los Tornos, 845 m asl; 06/VIII/2022; M.A. Marcos-García Leg.; CEUA00115263 · 1♀; Lugo, Negueira de Muñiz, Robledal; 22/III/2022; A. Uría Leg.; AUC · 1♂; Navarra, Itxaco, Isaba; 30/IV/1978; Unknown Leg.; UNAV (previously identified as *C. chloris*).

**Published. SPAIN ·** 9♂♂ and 5♀♀; León, Puerto El Pontón; 9/IX/1988; M.A. Marcos-García Leg.; CEUA00016735 to 00016741, CEUA00016745 to 00016751 · 1♂; León, Puerto Señales; 3/VI/1988; M.A. Marcos-García Leg.; CEUA00016742 · 1♂ and 1♀; León, Cofiñal; 09/IX/1988; M.A. Marcos-García Leg.; CEUA00016743 and 00016752 · 1♂; Asturias, Tielve; 10/IX/1988; M.A. Marcos-García Leg.; CEUA00016744 · 1♂; Cantabria, Fuente Dé; 11/IX/1988; M.A. Marcos-García Leg.; CEUA00016753 · 1♀; León, Hayedo de Pandetrave; 06/VI/1988; M.A. Marcos-García Leg.; CEUA00016754 (all previous specimens published in [29]) · **BOSNIA AND HERZEGOVINA** · 1♂; Jahorina; 26/VI/1989; A. Vujić Leg.; FSUNS (published in [12]) · **SERBIA ·** 1♀; Kopaonik, Velika Reka; 14/VI/1986; A. Vujić Leg.; FSUNS (published in [12]).

***Cheilosia brachysoma* Egger, 1860**

**Examined material. New. POLAND ·** 1♀; Bieszozady, nadl. Tarnawa del. Sanu k./pot. Mucineyo; 18/VII/1969; S. Bal Leg.; CEUA00016901.

*Cheilosia bracusi* Vujić and Claussen, 1994*

**Examined material. New. SPAIN ·** 18♂♂; Huesca, Valle de Otal, Bujaruelo, 1620 m asl, 42°41′28″ N 0°9′12″ W; 25/V/2023; I. Ballester-Torres Leg.; CEUA00115291 to 00115308 · 4♂♂ and 3♀♀; Huesca, Bujaruelo, Valle de Otal, 1600 m asl, 42°41′49″ N 0°7′47″ W; 01/VI/2024; Ballester-Torres and Orengo-Green Leg.; CEUA00116961 to 00116967 · 5♂♂ and 1♀; Huesca, Bujaruelo, Praderas de Laña Larga, 1360 m asl, 42°41′54″ N 0°6′41″ W; 01/VI/2024; Ballester-Torres and Orengo-Green Leg.; CEUA00116968 to 00116973 · 4♂♂ and 1♀; same locality and Leg. as previous; 02/VI/2024; CEUA00116974 to 00116978.

**Published. MONTENEGRO ·** 1♂; Bioč, Down lake (lake Stabansko), 43.184 18.729; 18/V/2017; Vujić et al. Leg.; FSUNS (published in [12]) · **SLOVENIA ·** 1♂; PARATYPE; Pokljuka; 22/V/1989; A. Vujić Leg.; FSUNS (published in [12]) · **NORTH MACEDONIA** · 1♀; PARATYPE; Baba; 8/V/1990; A. Vujić Leg.; FSUNS (published in [12]).

*Cheilosia carbonaria* Egger, 1860*

New to the Iberian Peninsula

**Figure 11:**
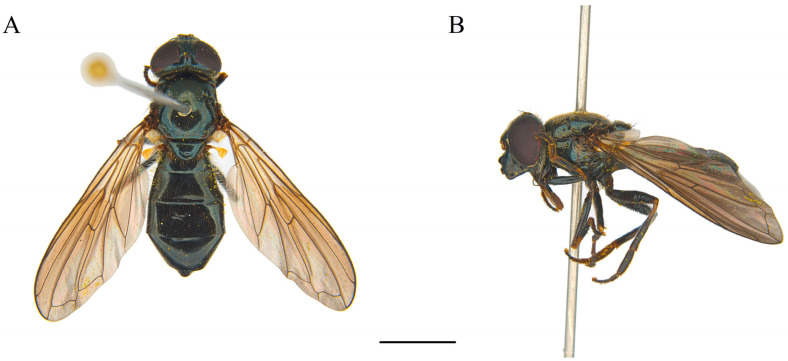
*Cheilosia carbonaria* from Sant Joan de Toran, Lleida, Spain. (**A**) Female, dorsal view. (**B**) Female, lateral view. Scale bar: (**A**,**B**) 2.5 mm.

**Examined material. New. SPAIN ·** 1♀; Lleida, Sant Joan de Toran, Refugi Honeria, 1060 m asl, 42° 49′11″N 0°48′19″ E; 18/VI/2024; Ballester-Torres, Quinto, Martínez-Pérez Leg.; CEUA00116960 (Figure 11A,B) · **POLAND** · 1♂; Bieszczady, Dwernik; 23/V/1963; R. Bankowska Leg.; CEUA00016755 · 1♀; Grotniki, Distr. Lódz; 14/V/1989; B. Sossynski Leg.; CEUA00016757 · **DENMARK** · 1♀; Grejsdalen; 26/VII/1962; E. Torp Leg.; CEUA00016756.

**Published. SLOVENIA** · 1♂; Savica; 14/VI/1988; A. Vujić Leg.; FSUNS (published in [12]) · **MONTENEGRO ·** 1♀; Durmitor, Luke; 30/VI/1985; A. Vujić Leg.; FSUNS (published in [12]).

*Cheilosia chloris* (Meigen, 1822)*

**Examined material. New. GERMANY ·** 1♀; Schleswig-Holst. Bremsberg NF2037 Mischwald; 16/VI/1979; Clauβen Leg.; CEUA00016911 · 1♂; Schlesw.—Holst. Langballigau; 14/V/1983; Clauβen Leg.; CEUA00016909.

**Published. SLOVENIA ·** 1♂; Pokljuka; 22/V/1989; A. Vujić Leg.; FSUNS (published in [12]).

*Cheilosia cynocephala* Loew, 1840*

New to the Iberian Peninsula.

**Figure 12:**
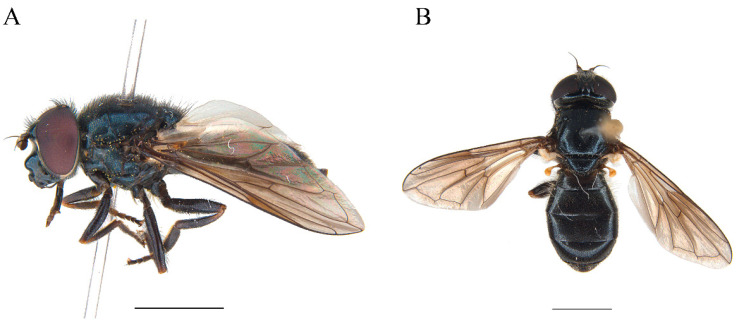
*Cheilosia cynocephala* from Portillo de Eraize, Navarra, Spain. (**A**) Male, lateral view. (**B**) Female, dorsal view. Scale bars: (**A**,**B**) 2.5 mm.

**Examined material. New. SPAIN ·** 3♂♂ and 4♀♀; Navarra, Portillo de Eraize, 1578 m asl; 19/VII/2022; A. Ricarte and Z. Nedeljković Leg.; CEUA00114743 to 00114749 (Figure 12A,B).

**Published. FRANCE ·** 1♀; Dorres, Les Garberes, 1560 m asl; 08/VI/2016; X. Lair Leg.; XLPC (published in [30]) · **SERBIA ·** 1♂; Obedska Bara, Debela Gora; 23/IV/1988; A. Vujić Leg.; FSUNS (published in [12]).

*Cheilosia fraterna* (Meigen, 1830)*

**Examined material. New. SPAIN ·** 1♂; Asturias, Soto de Aguas; 20/IV/2019; Piluca Álvarez Leg.; CEUA00109100 · 4♂♂ and 1♀; Huesca, Hayedo de Salenques, 1460 m asl, 42°35′49″ N 0°45′27″ E; 15/VI/2024; Ballester-Torres, Quinto, Martínez-Pérez Leg.; CEUA00116931 to 00116935 · 1♀; same locality and Leg. as previous; 16/VI/2024; CEUA00116936 · 1♂; same locality and Leg. as previous; 17/VI/2024; CEUA00116937 · 2♀♀; Lleida, Sant Joan de Toran, Refugi Honeria, 1060 m asl, 42°49′11″ N 0°48′19″ E; 18/VI/2024; Ballester-Torres, Quinto, Martínez-Pérez Leg.; CEUA00116938 and 00116939 · 1♀; Navarra, Bosque de Irati, Valle de Irati, Exp. Inst. Esp. Entomología; 02/VII/1947; Unknown Leg.; MNCN · 1♀; Madrid, Sierra de Guadarrama; 01/VI/1926; Dusmet Leg.; MNCN · 1♀; Navarra, Quinto Real; 10/VI/1984; T. Valdes Leg.; UNAV (previously identified as *C. chloris*) · 1♀; Lleida, Vall d’Arán, Montgarri; 18/VII/1992; MZB, Ex Col. Artigas; MCNB · 1♂; Barcelona, Sta. Fe, Montseny; 25/IV/1992; MZB, Ex Col. Artigas; MCNB.

**Published. SPAIN ·** 3♂♂ and 2♀♀; León, Brañillín; 02/VI/1988; M.A. Marcos-García Leg.; CEUA00016760 to 00016762, 00016771, and 00016772 · 1♂; León, Puerto Pajares; 04/VI/1987; M.A. Marcos-García Leg.; CEUA00016763 · 3 ♂♂; León, Puerto Leitariegos; 01/VI/1988; M.A. Marcos-García Leg.; CEUA00016764, 00016765, and 00016777 · 1♀; same locality as for the preceding; 13/VI/1986; M.A. Marcos-García Leg.; CEUA00016776 · 1♀; same locality as for the preceding; 02/VI/1987; M.A. Marcos-García Leg.; CEUA00016778 · 3♂♂; León, Aralla; 02/VI/1988; M.A. Marcos-García Leg.; CEUA00016766, 00016767, and 00016773 · 1♀; same locality as for the preceding; 03/VI/1987; M.A. Marcos-García Leg.; CEUA00016769 · 4♀♀; León, Alto del Pontón; 06/VII/1986; M.A. Marcos-García Leg.; CEUA00016770, 00016779 to 00016781 · 1♀; same locality as for the preceding; 12/IX/1987; M.A. Marcos-García Leg.; CEUA00016768 · 1♂; León, Cofiñal; 03/VI/1988; M.A. Marcos-García Leg.; CEUA00016774 · 1♀; León, Puerto de Pandetrave; 12/VI/1987; M.A. Marcos-García Leg.; CEUA00016775 (all previous specimens published in [31,32]) · **SLOVENIA ·** 1♂; Pokljuka; 22/V/1989; A. Vujić Leg.; FSUNS (published in [12]).

***Cheilosia lenis* Becker, 1894**

**Examined material. New. CZECH REPUBLIC ·** 1♂; Bohemia, Lestkov-Vel. Lesna, distr. Chomutov, sq. 5645, 500 m asl; 05/V/1996; L. Mazánek Leg.; CEUA00016961 · 1♀; Moravia, Nrubá voda.distr.olomus, sq. 6370, 350 m asl; 03/V/1993; L. Mazánek Leg.; CEUA00016960 · **FRANCE ·** 1♂; Nr. Chabreloche, lay-by on A710, Puy-de-Dôme, 700 m asl; 19/IV/2007; M.C.D. Speight Leg.; CEUA00115264 · 1♀; Ballon d’Alsace, Vosges; 19/VI/1979; M.C.D. Speight Leg.; CEUA00115265 **· POLAND ·** 1♀; Mts. Planiny; 05/V/196[illegible]; K. Malski Leg.; CEUA00016959.

**Published. SERBIA ·** 1♂ and 1♀; Kopaonik, Samokovska Reka-S; 22/V/1986; A. Vujić Leg.; FSUNS (published in [12]).

***Cheilosia lenta* Becker, 1894**

**Examined material. Published. GREECE ·** 1♂; Metsovo-Katara; 13/V/1990; A. Vujić Leg.; FSUNS (published in [12]) · **SERBIA ·** 1♀; Vršac, Dom-Brana; 25/IV/1986; A. Vujić Leg.; FSUNS (published in [12]).

***Cheilosia melanura* Becker, 1894**

**Examined material. SWITZERLAND ·** 1♂; Van d’en Haut, Valais, 1370 m asl; 4/VI/1999; M.C.D. Speight Leg.; CEUA00115266 · 1♀; Gletsch, Valais, 1800 m asl; 6/VI/1998; M.C.D. Speight Leg.; CEUA00115267.

**Published. FRANCE ·** 1♂; Formiguères, Marais, 1526 m asl; 20/V/2019; X. Lair Leg.; XLPC · 1♀; same locality as preceding; 23/VI/2019; X. Lair Leg.; XLPC · 1♀; same locality as preceding; 05/VI/2020; X. Lair Leg.; XLPC · 2♂♂ and 2♀♀; Formiguères, Refuge de la Jaceta; 16/V/2019; X. Lair Leg.; XLPC · 2♂♂ and 2♀♀; same locality as preceding; 30/V/2019; X. Lair Leg.; XLPC (all previous specimens published in [33]).

***Cheilosia rhynchops* Egger, 1860**

**Examined material. Published. SLOVENIA ·** 1♂; Menina-G; 24/V/1989; A. Vujić Leg.; FSUNS (published in [12]) · **MONTENEGRO ·** 1♀; Durmitor, Luke; 8/VII/1991; A. Vujić Leg.; FSUNS (published in [12]).

*Cheilosia vernalis* (Fallen, 1817)*

**Examined material. New. SPAIN** · 1♂; Asturias, Viesques, en *R. ficaria*; 22/III/2018; Piluca Álvarez Leg.; CEUA00109106 · 1♀; León, Posada de Valdeón, Santa Marina, 43°7′57.5″ N 4°53′19.9″ O, 1138 m asl; 24/VIII/2021; A. Ricarte and Z. Nedeljković Leg.; CEUA00111350 · 1♀; León, Posada de Valdeón, Pto. de Panderrueda, 43°7′31″ N 4°58′53.9″ O, 1463 m asl; 27/VIII/2021; A. Ricarte and Z. Nedeljković Leg.; CEUA00111352 · 1♀; León, Posada de Valdeón, cerca hotel Cumbres, 43°9′3″ N 4°55′9″ O, 923 m asl; 23/VIII/2021; A. Ricarte and Z. Nedeljković Leg.; CEUA00111354 · 2♂♂ and 1♀; León, Posada de Valdeón, Los Llanos, 43°9′23.6″ N 4°54′59″ O, 902 m asl; 27/VIII/2021; A. Ricarte and Z. Nedeljković Leg.; CEUA00111315, 00111319, and 00111353 · 1♂; Huesca, Guarrinza; 17/VIII/1991; M.A. Marcos-García Leg.; CEUA00109105 · 3♂♂; Navarra, Valle de Roncal, Mata de Haya; 18/VII/2022; A. Ricarte and Z. Nedeljković Leg.; CEUA00114740 to 00114742 · 1♀; Huesca, Torla-Ordesa, Orillas Río Ara, 965 m asl, 42°37′49″ N 0°6′29″ W; 21/V/2023; I. Ballester-Torres Leg.; CEUA00115309 · 1♂; Huesca, Prado cerca S. Nicolás Bujaruelo, 1335 m asl, 42°41′16″ N 0°6′25″ W; 23/V/2023; I. Ballester-Torres Leg.; CEUA00115310 · 1♂ and 1♀; Huesca, Parque Nacional de Ordesa y Monte Perdido, Pradera de Ordesa, 1310 m asl, 42°39′57″ N 0°3′33″ W; 23/V/2023; I. Ballester-Torres Leg.; CEUA00115311 and 00115312 · 1♀; Lugo, Balouta; 18/V/1986; M.A. Marcos-García Leg.; CEUA00017129 · 2♂♂ and 1♀; Huesca, Bujaruelo, Prederas de Laña Larga, 1360 m asl, 42°41′54″ N 0°6′41″ W; 01/VI/2024; Ballester-Torres and Orengo-Green Leg.; CEUA00116940 to 00116942 · 5♂♂ and 2♀♀; Huesca, Bielsa, Entrada al pueblo, Prado, 990 m asl, 42°37′50″ N 0°13′21″ E; 06/V/2024; I. Ballester-Torres Leg.; CEUA00116943 to 00116949 · 7♂♂ and 2♀♀; same locality and Leg. as previous; 07/V/2024; CEUA00116950 to 00116958 · 1♀; Lleida, Bausen, Hayedo de Carlac, 965 m asl, 42°50′14″ N 0°43′28″ E; 19/VI/2024; Ballester-Torres, Quinto, Martínez-Pérez Leg.; CEUA00116959.

**Published. SPAIN ·** 1♂; León, Alto del Pontón; 09/IX/1988; M.A. Marcos-García Leg.; CEUA00017122 · 1♂; same locality as previous; 12/IX/1987; M.A. Marcos-García Leg.; CEUA00017130 · 1♂; León, Beberinos; 11/IX/1987; M.A. Marcos-García Leg.; CEUA00017125 · 1♂ and 1♀; Cantabria (labelled as Santander), Vada; 12/IX/1988; M.A. Marcos-García Leg.; CEUA00017124 and 00017132 · 1♂; Cantabria (labelled as Santander), Desf. La Hermida; 10/XI/1986; M.A. Marcos-García Leg.; CEUA00017119 · 1♂; same locality as previous; 15/VII/1987; M.A. Marcos-García Leg.; CEUA00017120 · 1♂; León, Cofiñal; 09/IX/1988; M.A. Marcos-García Leg.; CEUA00016946 (published as *Cheilosia impressa*) · 1♀; León, Puerto de las Señales; 16/VI/1986; M.A. Marcos-García Leg.; CEUA00017133 · 1♀; León, Brañillín; 02/VI/1988; M.A. Marcos-García Leg.; CEUA00017134 · 1♀; Asturias, Santillán; 09/IX/1988; M.A. Marcos-García Leg.; CEUA00017135 (all specimens published in [31,32]) · 1♀; Salamanca, Béjar; 15/III/1977; M.A. Marcos-García Leg.; CEUA00017128 (published in [34]) · 1♀; Girona, Villalonga de Ter, orllas del Ter, cerca Ctra. Villalonga-Setcases, 1166 m asl; 2/VIII/2020; Z. Nedeljković Leg.; CEUA00108793 (published in [35]) · 1♂; Girona, Setcases, camino del depósito de agua, 1317 m asl; 31/VII/2020; Z. Nedeljković Leg.; CEUA00108795 (published in [35]) · **BOSNIA AND HERZEGOVINA ·** 1♂; Grmeč; 30/IV/1990; A. Vujić Leg.; FSUNS (published in [12]) **· SERBIA ·** 1♀; Vršac, Gudul. Vrh.; 2/V/1985; A. Vujić Leg.; FSUNS (published in [12]).

***Cheilosia* aff. *fraterna* sensu van Eck** [16,36]*

**Examined material. Published. PORTUGAL ·** 1♂; Medas, near Douro, UTM: 29T0546-4545, 40 m asl; 17/IV/2006; A.v. Eck Leg.; AVPC · 1♀; Palmeira de Faro, Quinta da Seara, UTM: 29T 521.5 4598.8, 60 m asl; 28/IV/2013; A.v. Eck Leg.; AVPC (all previous specimens published in [36]).

**Notes**. The taxonomic status of this species is still uncertain due to the availability of just a few specimens and the lack of DNA data.

### 3.4. Updated Checklist of the Iberian Species of the Cheilosia melanura Group

Following Ricarte and Marcos-García [14] and van Eck [15,16], after the revision of published and new materials of various species (see Section 3.3), an updated checklist of the species of the *C. melanura* group is provided here for the Iberian Peninsula (mainland Spain plus Portugal), excluding Andorra and Gibraltar. A total of 10 species, including one from Portugal of uncertain status, are listed in Table 2. Spain has nine species, while Portugal has three species recorded. Following [37], the IUCN Red List category is indicated for every species assessed in Europe/EU27.

### 3.5. Key to the Iberian Species of the Cheilosia melanura Group

Males and females

**Figure 13:**
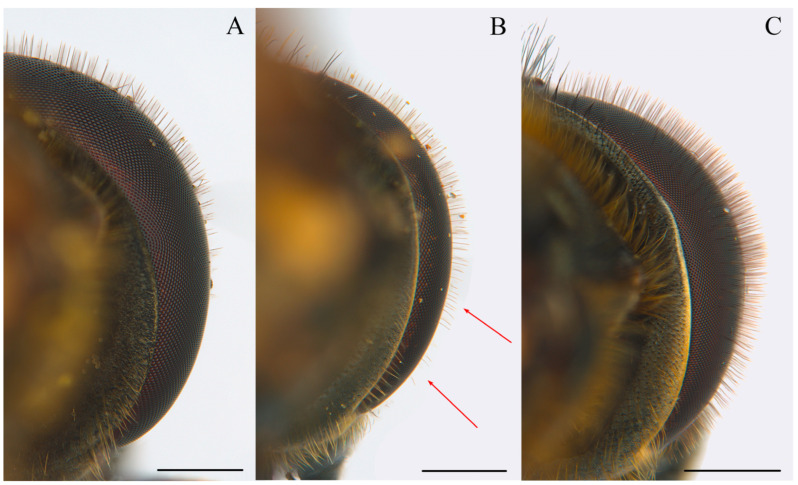
Heads of species of the *Cheilosia melanura* group in posterior view. (**A**). *Cheilosia bergenstammi*, male (**B**). *Cheilosia triamilia* sp. nov., male. Red arrows indicate the presence of hairs (**C**). *Cheilosia bracusi,* male. Scale bars: (**A**,**B**) 0.5 mm; (**C**) 0.75 mm.

**Figure 14:**
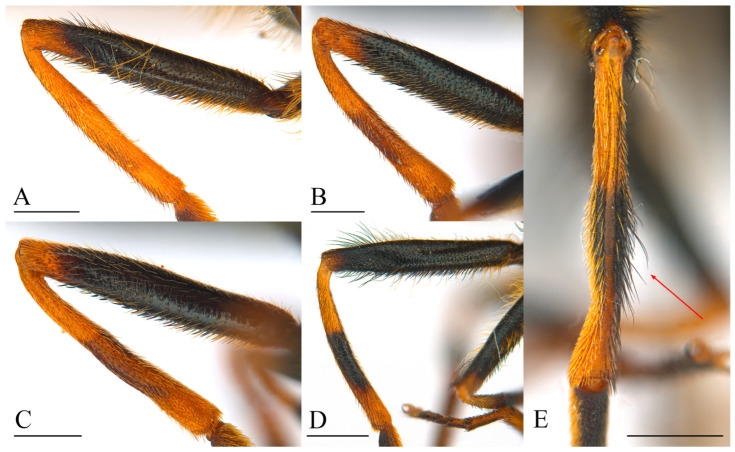
Hind legs of species of the *Cheilosia melanura* group to show the colour and pilosity of the tibia, anterior view (except for (**C**), posterior view; (**E**), dorsal view) (**A**). *Cheilosia fraterna,* male. (**B**). *Cheilosia triamilia* sp. nov., female. (**C**). *Cheilosia triamilia* sp. nov., female. (**D**). *Cheilosia bracusi,* male. (**E**). *Cheilosia bracusi,* male. Red arrows indicate the presence of long hairs. Scale bars: (**A**,**E**) 0.75 mm; (**B**,**C**) 0.5 mm; (**D**) 1 mm.

**Figure 15:**
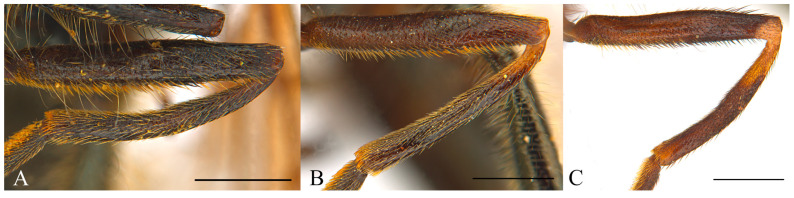
Hind legs of *Cheilosia andalusiaca,* highlighting the variability in the tibia colour*,* anterior view. (**A**). Male with a completely black tibia. (**B**). Female with a basally orange tibia. (**C**). Male with an orange tibia at both ends. Scale bars: (**A**–**C**) 0.75 mm.

1. Wing with a circular dark spot in the middle, sometimes faintly pigmented (Figure 11A and Figure 12B)……………………………………………………………………………**2**
  Wing hyaline or with a different pattern of pigmentation…………………………………………………………………………………………………………………………………...**3**
2. Body with blueish sheen. Basoflagellomere dark, arista at most 2.5 x the basoflagellomere height. Males: mesonotum hairs predominantly black, erect. Females: mesonotum hairs black, short, erect, abdomen widening up to posterior part of tergum II (Figure 12A,B)………………………………………………..***Cheilosia cynocephala***
  Body without blueish sheen. Basoflagellomere reddish-brown, arista at least 3 x the basoflagellomere height. Males: mesonotum hairs long predominantly pale, erect, longer black hairs intermixed in posterior part of mesonotum. Females: mesonotum hairs pale, short, semi-adpressed, abdomen widening up to posterior part of tergum III, then narrower in tergum IV and V (Figure 11A,B) ………………………………………………………………………………………………………………***Cheilosia carbonaria***
3. Males: eyes holoptic (connected) ………………………………………………………………………………………………………………………………………………………………**4**
  Females: eyes dichoptic (separated) …………………………………………………………………………………………………………………………………………………………..**10**
4. Eye on the lower half bare or with isolated hairs (Figure 13A)……………………………………………………………………………………………………………………………..**5**
  Eye hairy all over the surface, sometimes sparse on the lower half, with at least some hairs on the lowest part, without bare appearance (Figure 13B,C)…………………..**6**
5. Hind tibia completely pale or with a faint incomplete black ring (Figure 14A–C)…………………………………………………………………………………..***Cheilosia fraterna***
  Hind tibia with a complete black ring centrally (Figure 14D)…………………………………………………………………………………………………..***Cheilosia bergenstammi***
6. Hind tibia completely black, sometimes with pale basal part or rarely both ends pale (Figure 15A–C)…………………………………………………….***Cheilosia andalusiaca***
  Hind tibia completely pale, with an incomplete or a complete black ring centrally (Figure 14A–D)…………………………………………………….…………………………....**7**
7. Hind tibia with distinct long black hairs on the anterior surface (Figure 14E)…………………………………………………………………………..…………..***Cheilosia bracusi***
  Hind tibia without distinct long hairs on the anterior surface……………………………………………………………………………………………………………………………..**8**
8. Basal part of the arista yellow……………………………………………………………………………………………………………………………………………….***Cheilosia chloris***
  Basal part of the arista black…………………………………………………………………………………………………………………………………………………………………....**9**
9. Eye uniformly hairy. Posterior part of scutellum with thick black bristles (very rarely without), hind tibia with a black ring centrally (Figure 13C and Figure 14D)……………………………………………………………………………………………………………………………………………………………………………***Cheilosia vernalis***
  Eye sparse-haired on lower half. Posterior part of scutellum without distinct black bristles, hind tibia with a ventrally incomplete black ring (rarely complete) (Figure 13B and Figure 14B,C) ………………………………………… ………………………………………………… ………………………………………………… ..***Cheilosia triamilia* sp. nov.**
10. Eye on the lower half bare or with isolated hairs (Figure 13A)……………………………………………………………………………………………………………………………**11**
  Eye hairy all over the surface, sometimes sparse on the lower half, with at least some hairs on the lowest part, without bare appearance (Figure 13B,C)………………….**13**
11. All tibiae reddish pale, sometimes with a very faint dark spot centrally (Figure 14A) ……………………………………………………………………………..***Cheilosia fraterna***
  All tibiae with a conspicuous black ring, sometimes incomplete, but evident (Figure 14B–D)………………………………………………………………………………………..**12**
12. Basoflagellomere shiny orange, enlarged, approximately 3 x the parafacia width. Posterior part of scutellum with thick black bristles…………….***Cheilosia bergenstammi***
  Basoflagellomere dark, sometimes with reddish basal part, not enlarged, approximately 2 x the parafacia width. Posterior part of scutellum without distinct black bristles (see also couplet 15) …………………………………………………………………………………………………………………………………….***Cheilosia triamilia* sp. nov.**
13. Hind tibia completely black, sometimes with pale basal part or rarely both ends pale (Figure 15 A–C) ……………………………………………………***Cheilosia andalusiaca***
  Hind tibia completely pale, with an incomplete or a complete black ring centrally (Figure 14A–D) ………………………………………………………………………………..**14**
14. Posterior part of scutellum with thick black bristles. Hind tibia with a complete black ring centrally (Figure 14D)……………………………………………***Cheilosia vernalis***
  Posterior part of scutellum without distinct black bristles. Hind tibia pale or with an incomplete black ring centrally (Figure 14A–C)………….……….……………………**15**
15. Small species (7–9 mm approx.). Basoflagellomere black, sometimes dark brown with a reddish basal part………………………………………..***Cheilosia triamilia* sp. nov.**
  Larger species (>10 mm approx.). Basoflagellomere completely shiny or dull orange, sometimes the apical part darker…………………………………………………………**16**
  Arista shiny pale-coloured basally …………………………………………………………………………………………………………………………………………..***Cheilosia chloris***
16. Arista completely dark, non-shiny neither pale basally…………………………………………………………………………………………………………………..***Cheilosia bracusi***

## 4. Discussion

After the present work, the number of species of the *C. melanura* group that are present in the Iberian Peninsula doubled from 5 to 10, including a new species (*C. triamilia* sp. nov.), a new addition to the group (*C. andalusiaca*), two new species for Spain (*C. carbonaria* and *C. cynocephala*), and a taxonomically unresolved species from Portugal (*C.* aff. *fraterna*). The present study is the first to morphologically revise the *C. melanura* group in combination with molecular evidence and to provide a key to some of its European species.

In some of the published keys to *Cheilosia* species, *C. triamilia* sp. nov. would key out as *C. bergenstammi*, *C. fraterna, C. vernalis,* or even *Cheilosia alpina* (Zetterstedt, 1838) [11,12,38]. *Cheilosia alpina* belongs to a different species group, as it has longer hairs than *C. triamilia* sp. nov. and a totally different morphology of the male genitalia, including a very characteristic ventral lobe of the surstylus [21]. Dichotomous keys of the species of the *C. melanura* group were not available, as some of the known keys are based on the keys of Sack [39]. These discrepancies while identifying our new species are due to the morphological traits selected by Sack, as some of these are variable in individuals of the species in the *C. melanura* group. Variable characteristics include the presence or absence of bristles in the posterior part of the scutellum or the presence or absence of eye hairs in females [3,38]. *Cheilosia triamilia* sp. nov. resembles a large *C. vernalis*, except for the uniformity of the eye hairs (entirely uniformly hairy in *C. vernalis* but with sparser hairs on the basal half of the eye in *C. triamilia* sp. nov.), the absence of bristles in the posterior part of the scutellum (present in *C. vernalis*), and the hind tibiae with an incomplete or faint black ring centrally (always complete and entirely black in *C. vernalis*). Regarding the similarity of the new species with *C. bergenstammi* and *C. fraterna*, according to the pattern of the eye hairs, both *C. bergenstammi* and *C. fraterna* have no hairs on the basal half of the eye (sometimes with some isolated hairs, but the lowest part is always bare) (Figure 13A), whilst in *C. triamilia* sp. nov., the eye is entirely hairy, with sparser hairs on the basal half, but always with at least some hairs on the lowest part (Figure 13B). *Cheilosia bergenstammi* has a bright yellowish antenna, which is enlarged in females but completely dull and dark in *C. triamilia* sp. nov., and the females have a basoflagellomere of the same size as that of males, showing a reddish tonality but never appearing to be shiny or occupying the entire surface. Regarding *C. fraterna*, both males and females have a basoflagellomere of the same size, and it is completely orange and sometimes dull, without darker parts. The hind tibiae of the males of *C. triamilia* sp. nov. are mainly pale or have an incomplete black ring (Figure 14B,C), as in *C. fraterna* (Figure 14A) (rarely complete in *C. triamilia* sp. nov.), while the hind tibiae of *C. bergenstammi* have a complete black ring centrally (Figure 14D).

The ML consensus tree of the COI-5′ sequences shows differences between the known species of *C. melanura* and our new species from the Sierra Nevada, with a high bootstrap value (Figure 3). The analysis of the COI-5′ sequences revealed that *C. triamilia* sp. nov. is closely related to *C. fraterna*, as both are included in a polytomy together with some other species of the group. The observed polytomy underlines the genetic complexity of the group, as has already been shown in other publications [3], highlighting that phylogenetic analyses with more than one molecular marker are needed to disentangle the systematics of the *C. melanura* group, although some species such as *C. bergenstammi, C. carbonaria,* and *C. triamilia* sp. nov. are well differentiated with only one marker (Figure 3).

Torp Pedersen [40] described a new species of *Cheilosia, C. andalusiaca*, which was also from the surrounding areas of the Sierra Nevada National Park. Since then, *C. andalusiaca* has been found in a couple of high-altitude mountains in Spain with a few records [14,41]. After the morphological revision of several specimens, we concluded that they shared the typical morphology of the group, including the male genitalia (Figure 10). Unfortunately, we were only able to amplify the ‘C’ fragment of the COI-5′ sequence of *C. andalusiaca*, but the ML tree revealed that the species is grouped with the *C. melanura* group, as it is closely genetically related to *C. rhynchops* and *C. lenis* (Figure 4). Due to the scarce citations of the species since its description and its similarity to other species, such as *C. lenta* and *C. rhynchops*, the taxonomic status of *C. andalusiaca* remained unknown. The mesonotum of male *C. rhynchops* is completely covered in black hairs, whereas male *C. andalusiaca* has white hairs on the entire mesonotum surface. Females of *C. rhynchops* have short, adpressed hairs on the mesonotum, while in *C. andalusiaca,* the mesonotum hairs are longer and erect. On the other hand, *C. lenta* males have entirely dark legs, whilst *C. andalusiaca* has paler tibia bases. Although the face of *C. lenta* is rather shiny, *C. andalusiaca* has a slightly white-dusted face, which is more conspicuous just beneath the antennal insertions. Mesonotum hairs in *C. lenta* females are short and semi-adpressed, whilst in *C. andalusiaca,* these hairs are longer and erect. *Cheilosia lenta* has a sternum II with short, erect hairs, but these hairs are longer in *C. andalusiaca.* As there were no available sequences of *C. lenta,* we were unable to test its genetic resemblance with *C. andalusiaca*. Regarding the morphology of the genitalia, the main difference lies in the dorsal lobe, which is narrow and somewhat longer in *C. lenta* than in *C. andalusiaca*, in which it is wider and slightly shorter (Figure 10). The genitalia of the *C. melanura* group are rather similar among species, and it is sometimes difficult to use them as a defining morphological characteristic [12]. Regarding females of the *C. melanura* group, the difficulties in correctly identifying some of the species are well known by authors, as many of the species are extremely variable in their morphology (e.g., [3]). The problem may be resolved by capturing the corresponding males in the same locality and on the same date as the undetermined females. Given all of the evidence, we decided to include *C. andalusiaca* as a new member of the *C. melanura* group. New studies are needed to unravel the current distribution and population trend of this species, as only a few individuals have been collected since the 1980s.

Regarding the new citations of *Cheilosia* in the Iberian region, this is the first time that *C. carbonaria* and *C. cynocephala* have been recorded in Spain and the Iberian Peninsula. *Cheilosia cynocephala* has been reported in Central France [42] and the French Pyrenees [30], making its occurrence in the Spanish Pyrenees likely. This is the first time that *C. carbonaria* has been reported in the Pyrenees. In 2018, *C. rhynchops* was misidentified as *C. carbonaria* in the French Pyrenees, but this misidentification was corrected before the publication of the manuscript [30]. However, as the species has already been reported in mainland France [43], the presence of this species in the northern region of the Iberian Peninsula was expected. Furthermore, in 2013, André van Eck collected a female in Portugal and identified it as *C. bracusi* [16]. Further studies of the specimen, together with a male that was also initially identified as *C. bracusi,* revealed that they belonged to a different species. Both individuals were labelled as *C.* aff. *fraterna* due to the possibility of this species being new and undescribed [36]. After a preliminary morphological and molecular study, the specimens of *C.* aff. *fraterna* appear to belong to an unnamed species resembling *C. brachysoma* and *Cheilosia siciliana* Becker, 1894. *Cheilosia* aff. *fraterna* has hairs all over the eyes, whilst *C. fraterna* always has the lowest part of the eyes completely bare. *Cheilosia* aff. *fraterna* is also similar to *C. triamilia* sp. nov.*,* but the general body colour is paler than that of *C. triamilia* sp. nov., and the basoflagellomere in *C.* aff. *fraterna* is completely pale orange. As there were no available sequences of *C. brachysoma* and *C. siciliana*, without a sufficient number of specimens of *C.* aff. *fraterna*, further studies are needed to confirm the identities of both specimens.

*Cheilosia triamilia* sp. nov. is the southernmost species of the *C. melanura* group, along with *C. andalusiaca*, both with close type localities in Sierra Nevada. *Cheilosia triamilia* sp. nov. was collected in several localities of the Sierra Nevada National Park, all above an altitude of 1800 m. This may show a dependence of the species on the high-altitude ecosystems of the park, which are popularly called “borreguiles”. The discovery of an isolated species of the *C. melanura* group in the southern part of the Iberian Peninsula agrees with the existence of glacial refugia during the Pleistocene period in the Iberian region [44], a hypothesis that is strongly supported by 65 endemic animal and plant species that exclusively inhabit the Sierra Nevada mountains [45]. For example, the distribution of *Baetica ustulata* (Rambur, 1838) (Orthoptera: Tettigoniidae) is also restricted to an altitudinal range above 2200 m in this mountainous system, which is associated with xeric biotopes with thorny cushioned vegetation [46]. *Cheilosia triamilia* sp. nov. may be a product of this Pleistocene speciation, and because of its limited distribution, more studies are necessary to determine the population size and its conservation status.

This distribution restricted to the high-altitude ecosystems of the Sierra Nevada could also be critical in the current climate change scenario, as some studies determined that high-mountain species of *Cheilosia* and other genera were model-predicted to lose their climatically suitable areas [47,48]. For example, Rotheray and Gilbert [49] predicted the extinction of *Cheilosia sahlbergi* (Becker, 1894) in the United Kingdom by 2080. This species, similarly to almost all members of the subgenus *Taeniocheilosia* Oldenberg, is exclusively montane, as that is where suitable habitats can be found [50]. The similarities in the altitudinal distribution between the *Taeniocheilosia* species and the new species described in this paper lead us to believe that climate change could affect *C. triamilia* sp. nov. in the same way as it may with *C. sahlbergi* or other montane hoverflies. According to the literature [14] and the new citations given in this work, all species of the *C. melanura* group are closely related to high-altitude ecosystems, so the mentioned climate change scenarios may influence them in the same way that was predicted for *C. sahlbergi*. However, some of the *C. fraterna* individuals found in Portugal were caught in lowlands [15], in addition to a male near 400 m asl in Asturias, Spain (see the additional examined material). The north of the Iberian Peninsula has similar climatic features to those of central and western Europe [51], where various species of the *C. melanura* group are also known to inhabit lowlands [6]. Due to this wider altitudinal range, the possible responses to climate change scenarios for the species of the *C. melanura* group may be somewhat unpredictable, varying from those obtained for *C. sahlbergi*. As climate change has been proven to be the main cause of biodiversity loss in high-altitude ecosystems [52], in situ conservation efforts may be insufficient for preserving the endemic species that are closely linked to the high-altitude ecosystems of the Iberian mountains. Ex situ breeding and conservation programs, such as those conducted for the syrphid *Blera fallax* (Linnaeus, 1758) in the UK [53,54], can be helpful for the conservation of species of the *C. melanura* group that have their habitats threatened by climate change, but the implementation of these programs requires extensive knowledge of the biology of these species as both adults and in the larval stages. Another recommended action for the conservation of these and other hoverfly species could be to create an Iberian Red List of Syrphidae that is complementary to the one developed in 2021 at the European/EU27 level [37]. For this purpose, future studies are needed in order to improve the knowledge of the range and biology of *C. triamilia* sp. nov., as well as to try to identify possible threats to its survival (activity of a ski resort in the Sierra Nevada, etc.).

## Figures and Tables

**Table 1:** GenBank and/or BOLD accession numbers for the gene sequences used in this study. ‘CEUA’ sequences are new (except for CEUA_S110).

DNA Vouchers	Species	Locality and Date	Marker	GenBank Accessions	BOLD Accessions
CEUA_S408	*Cheilosia* *andalusiaca*	Spain, Granada, Capileira, 17/IV/2023	COI-5′ (“C” Fragm.)	PP982689	-
BC-ZSM-DIP-24155-D06	*Cheilosia * *bergenstammi*	Germany, Oytal, Seewaende, 05/VII/2014	COI-5′	-	AMTPD4602-16
BC-ZSM-DIP-24155-F12	*Cheilosia * *bergenstammi*	Germany, Oytal, Oybach E Oytalhaus, 16/VI/2014	COI-5′	-	AMTPD4632-16
CEUA_S402	*Cheilosia * *bergenstammi*	Spain, León, Posada de Valdeón, Puerto de Panderrueda, 27/VIII/2021	COI-5′	PP982677	-
CEUA_S403	*Cheilosia * *bergenstammi*	Spain, León, Posada de Valdeón, Puerto de Panderrueda, 27/VIII/2021	COI-5′	PP982676	-
CEUA_S362	*Cheilosia* *bracusi*	Spain, Huesca, Pyrenees, Valle de Otal, 25/V/2023	COI-5′	PP982682	-
CEUA_S363	*Cheilosia* *bracusi*	Spain, Huesca, Pyrenees, Valle de Otal, 25/V/2023	COI-5′	PP982681	-
BC-ZSM-DIP-24155-F10	*Cheilosia * *carbonaria*	Germany, Oytal, Oybach E Oytalhaus, 16/VI/2014	COI-5′	-	AMTPD4630-16
jka06-02413	*Cheilosia * *carbonaria*	Finland, Lilla Kummelberget, 15/VII/2006	COI-5′	OK065484	FIDIP2486-12
BC-ZSM-DIP-24155-D08	*Cheilosia * *chloris*	Germany, Oytal, Seewaende, 05/VII/2014	COI-5′	-	AMTPD4604-16
BC-ZSM-DIP-24155-F11	*Cheilosia * *chloris*	Germany, Oytal, Oybach E Oytalhaus, 16/VI/2014	COI-5′	-	AMTPD4631-16
jka12-0439	*Cheilosia * *cynocephala*	Finland, Helsinki, Vuosaaren mkp, 25/VI/2002	COI-5′	MZ627494	FIDIP2498-12
CEUA_S366	*Cheilosia * *cynocephala*	Spain, Navarra, Portillo de Eraize, 19/VII/2022	COI-5′	PP982688	-
CEUA_S367	*Cheilosia* *cynocephala*	Spain, Navarra, Portillo de Eraize, 19/VII/2022	COI-5′	PP982687	-
CEUA_S368	*Cheilosia * *cynocephala*	Spain, Navarra, Portillo de Eraize, 19/VII/2022	COI-5′	PP982686	-
CEUA_S369	*Cheilosia * *cynocephala*	Spain, Navarra, Portillo de Eraize, 19/VII/2022	COI-5′	PP982685	-
GBDP1298-06	*Cheilosia * *fraterna*	Germany	COI-5′	AY533362	-
BMNH(E)#1731728	*Cheilosia* *fraterna*	England, 30/VII/2015	COI-5′	-	BEEEE358-16
jka06-00461	*Cheilosia * *fraterna*	Finland, Paeivaerinne, 09/VI/2006	COI-5′	OK065433	FIDIP2500-12
NORSY462	*Cheilosia * *fraterna*	Norway, Asaktoppen, 01/VI/2010	COI-5′	-	NORSY462-15
GBDP1283-06	*Cheilosia* *lenis*	“Yugoslavia”	COI-5′	AY533347	-
CEUA_S352	*Cheilosia melanura*	France, Collet d’Anterne, 23/VI/2014	COI-5′	PP982684	-
CEUA_S355	*Cheilosia melanura*	France, Le Lignon, 24/VI/2014	COI-5′	PP982683	-
BC-ZSM-DIP-24052-G03	*Cheilosia melanura*	Germany, Oytal, Oybach E Oytalhaus, 01/VI/2014	COI-5′	-	AMTPD4540-16
BC-ZSM-DIP-24155-D07	*Cheilosia melanura*	Germany, Oytal, Seewaende, 05/VII/2014	COI-5′	-	AMTPD4603-16
NORSY299	*Cheilosia * *reniformis*	Norway, Ringerike, Busund, 16/VI/2009	COI-5′	-	NORSY299-12
jka07-04715	*Cheilosia * *reniformis*	Finland, Nylandia, Vantaa, Vehkalanmaeki, 19/IV/2007	COI-5′	MZ622955	-
jka07-04731	*Cheilosia * *reniformis*	Finland, Nylandia, Helsinki, Kivihaka, 22/IV/2007	COI-5′	MZ623785.1	-
BC-ZSM-DIP-24053-G08	*Cheilosia * *rhynchops*	Germany, Schochen, 21/VI/2014	COI-5′	-	AMTPE1125-15
jka07-06440	*Cheilosia * *sootryeni*	Finland, Luotteispuro, 04/VI/2007	COI-5′	MZ627393	FIDIP2514-12
jka07-06909	*Cheilosia * *sootryeni*	Finland, Kummelberget, 24/V/2007	COI-5′	MZ623648	FIDIP2517-12
CEUA_S110	*Cheilosia soror*	Spain, Alicante, Sierra de Aitana, 29/V/2020	COI-5′	ON097149	-
CEUA_S122	*Cheilosia * *triamilia* sp. nov.	Spain, Granada, Sierra Nevada, 25/VI/2021	COI-5′	PP982678	-
CEUA_S181	*Cheilosia * *triamilia* sp. nov.	Spain, Granada, Sierra Nevada, 27/V/2022	COI-5′	PP982679	-
CEUA_S182	*Cheilosia * *triamilia* sp. nov.	Spain, Granada, Sierra Nevada, 26/V/2022	COI-5′	PP982680	-
lampe00048	*Cheilosia * *vernalis*	Finland, Oulu, 08/VII/2010	COI-5′	JN269868	DIFIA048-11
MZH_HP.111	*Cheilosia* *vernalis*	Finland, Perkko, 22/V/2011	COI-5′	MZ631213	FIDIP111-11
MZH_HP.133	*Cheilosia * *vernalis*	Finland, Vasarankylae, 29/V/2011	COI-5′	OK065358	FIDIP133-11
BC-ZSM-DIP-24052-F10	*Ferdinandea * *cuprea*	Germany, Oytal, 01/VI/2014	COI-5′	-	AMTPD4535-16

**Table 2:** List of the Iberian species of the *Cheilosia melanura* group according to the present taxonomic revision. The Iberian state in which each species was reported, as well as the Spanish province, is specified in the second column. Spanish provinces where a species was reported for the first time are listed in bold. The IUCN Red List category in Europe/EU27 is also given in the third column according to the European Red List of Hoverflies. Legend: Barcelona (B), Vizcaya (BI), Cáceres (CC), Girona (GI), Granada (GR), Huesca (HU), Jaén (J), Lleida (L), León (LE), Lugo (LU), Madrid (M), Navarra (NA), Asturias (O), Santander (S), Salamanca (SA), Soria (SO), Guipúzcoa (SS), Álava (VI), Zaragoza (Z), Endangered (EN), Least Concern (LC).

Species	Distribution	IUCN Red List Category
*Cheilosia andalusiaca*	Spain: CC, GR, **J**, LE, O, SA,	EN
*Cheilosia bergenstammi*	Spain: **BU**, LE, **LU**, O, S, VI	LC
*Cheilosia bracusi*	Spain: **GI**, HU, **LE**	LC
*Cheilosia carbonaria*	Spain: **L**	LC
*Cheilosia chloris*	Spain: B, BI, LE, Z	LC
*Cheilosia cynocephala*	Spain: **NA**	LC
*Cheilosia fraterna*	Portugal/Spain: B, GI, **HU**, **L**, LE, LU, **M**, **O**, S	LC
*Cheilosia triamilia* sp. nov.	Spain: **GR**	-
*Cheilosia vernalis*	Portugal/Spain: B, BI, GI, **HU**, **L**, LE, LU, M, **NA**, O, S, SA, SE, SO, SS,	LC
*Cheilosia* aff. *fraterna*	Portugal	-

## Data Availability

All sequences generated in this work are available in the publicly accessible repository of GenBank (https://www.ncbi.nlm.nih.gov/genbank/) (accessed on 24 August 2024). Zoobank: urn:lsid:zoobank.org:act:80468FDD-8508-48F0-9EE6-390596590A94.

## Supplementary Materials

The following supporting information can be downloaded at: https://www.mdpi.com/article/10.3390/insects15090640/s1, Table S1: List of examined materials in CSV format.
